# Effect of a low-protein diet during mid-to-late gestation on reproductive performance and serum amino acid profiles in sows

**DOI:** 10.1093/jas/skaf412

**Published:** 2025-11-29

**Authors:** Qipeng Zhang, Zhongyu Liu, Guangrong Xie, Zhengfeng Fang, Lianqiang Che, Yan Lin, Shengyu Xu, Yong Zhuo, Lun Hua, Jian Li, Xuemei Jiang, Guangmang Liu, Ruinan Zhang, Mengmeng Sun, Min Yang, De Wu, Bin Feng

**Affiliations:** Key Laboratory of Animal Disease-Resistant Nutrition of Ministry of Education, Sichuan Agricultural University, Chengdu, Sichuan 611130, China; Animal Nutrition Institute, Sichuan Agricultural University, Chengdu, Sichuan 611130, China; Key Laboratory of Animal Disease-Resistant Nutrition of Ministry of Education, Sichuan Agricultural University, Chengdu, Sichuan 611130, China; Animal Nutrition Institute, Sichuan Agricultural University, Chengdu, Sichuan 611130, China; Key Laboratory of Animal Disease-Resistant Nutrition of Ministry of Education, Sichuan Agricultural University, Chengdu, Sichuan 611130, China; Animal Nutrition Institute, Sichuan Agricultural University, Chengdu, Sichuan 611130, China; Key Laboratory of Animal Disease-Resistant Nutrition of Ministry of Education, Sichuan Agricultural University, Chengdu, Sichuan 611130, China; Key Laboratory of Animal Disease-Resistant Nutrition of Ministry of Education, Sichuan Agricultural University, Chengdu, Sichuan 611130, China; Key Laboratory of Animal Disease-Resistant Nutrition of Ministry of Education, Sichuan Agricultural University, Chengdu, Sichuan 611130, China; Key Laboratory of Animal Disease-Resistant Nutrition of Ministry of Education, Sichuan Agricultural University, Chengdu, Sichuan 611130, China; Key Laboratory of Animal Disease-Resistant Nutrition of Ministry of Education, Sichuan Agricultural University, Chengdu, Sichuan 611130, China; Key Laboratory of Animal Disease-Resistant Nutrition of Ministry of Education, Sichuan Agricultural University, Chengdu, Sichuan 611130, China; Key Laboratory of Animal Disease-Resistant Nutrition of Ministry of Education, Sichuan Agricultural University, Chengdu, Sichuan 611130, China; Key Laboratory of Animal Disease-Resistant Nutrition of Ministry of Education, Sichuan Agricultural University, Chengdu, Sichuan 611130, China; Key Laboratory of Animal Disease-Resistant Nutrition of Ministry of Education, Sichuan Agricultural University, Chengdu, Sichuan 611130, China; Key Laboratory of Animal Disease-Resistant Nutrition of Ministry of Education, Sichuan Agricultural University, Chengdu, Sichuan 611130, China; College of Science, Sichuan Agricultural University, Ya’an, Sichuan 625014, China; Pet Nutrition and Health Research Center, Chengdu Agricultural College, Chengdu 611130, China; Key Laboratory of Animal Disease-Resistant Nutrition of Ministry of Education, Sichuan Agricultural University, Chengdu, Sichuan 611130, China; Animal Nutrition Institute, Sichuan Agricultural University, Chengdu, Sichuan 611130, China; Key Laboratory of Animal Disease-Resistant Nutrition of Ministry of Education, Sichuan Agricultural University, Chengdu, Sichuan 611130, China; Animal Nutrition Institute, Sichuan Agricultural University, Chengdu, Sichuan 611130, China

**Keywords:** amino acids, low-protein diet, nitrogen excretion, reproductive performance, sows

## Abstract

Low-protein (LP) diets have been proposed as a strategy for animal production to conserve protein resources and reduce environmental nitrogen pollution. The aim of this study was to investigate the effect of a LP diet during gestation on the reproductive performance, serum amino acid concentrations, and nitrogen excretion in sows. A total of 24 healthy Landrace × Yorkshire sows (4 to 5 parities) with similar body weight and back fat were fed with normal gestational diet (control group, crude protein = 13.65%, *n* = 12) or a LP gestational diet (LP group, crude protein = 11.30%, *n* = 12) from day 30 of gestation to the day before parturition. All sows received same diet during lactation. Results showed that LP diet during mid-to-late gestation reduced the gain of backfat thickness during days 30 to 60 of gestation (*P *< 0.05) compared with the control group, without changing the reproductive performance of the sows. The gene expression levels of amino acid transporters in the placenta, and the organ indices of neonatal and weaned piglets, were all unchanged by the LP diet. The digestion and metabolism experiment revealed that the LP diet decreased nitrogen excretion and net nitrogen deposition during late gestation (*P *< 0.05). Additionally, the LP diet reduced serum concentrations of valine, arginine, lysine, and methionine on day 110 of gestation, and arginine, lysine, and methionine on day 21 of lactation, while it tended to decrease serum concentration of the total essential amino acids and the level of threonine in the milk throughout lactation (*P *< 0.05). Gestational LP diet al.o significantly reduced concentrations of serine and the total non-essential amino acids in the serum of neonatal piglets (*P *< 0.05) and serum threonine in weaned piglets, but increased leucine (*P *< 0.05) in the serum of weaned piglets. In conclusion, an appropriate reduction of dietary protein during mid-to-late gestation in sows (11.30% vs. 13.65%) could reduce their nitrogen excretion while maintaining reproductive performance, and it is recommended to supplement arginine and valine in the LP diets in addition to lysine, methionine, tryptophan, and threonine. This study highlights the significance of low-protein diets in protein feed saving and environmental protection.

## Teaser

The current study demonstrates that an appropriate reduction of dietary protein in pregnant sows does not compromise reproductive performance while significantly reducing nitrogen excretion. Furthermore, we recommend supplementing the low-protein diets with valine, arginine, lysine, methionine, tryptophan, and threonine during gestation.

## Introduction

The demand for protein-rich feed ingredient is increasing with the continuous growth of the pig industry, while the costs of feeding and environmental pollution of nitrogen excretion have become urgent issues (Wang et al., 2018). Many studies have explored the use of alternative feedstocks other than soybean meal to reduce farming costs (Anderson et al., 2021; Cheng et al., 2022; Ibagon et al., 2023). However, these studies did not address the problem of excessive nitrogen excretion, and some alternative feedstocks contain many anti-nutritional factors.

Low-protein (LP) diets are formulated based on the theory of balanced amino acid nutrition. This approach involves reducing the dietary crude protein level by 2–4 percentage points relative to the NRC (2012) recommendations and supplementing with crystalline amino acids to precisely meet animal requirements. Such diets effectively lower nitrogen excretion without compromising animal health or production performance (Wang et al., 2018; Cappelaere et al., 2021; de Almeida et al., 2024). Though some studies have focused on protein-restricted diets during gestation in sows, it remains unclear what effect of LP diets on the reproductive performance and serum concentrations of amino acids in gestational sows (Rehfeldt et al., 2012; Yang et al., 2021; de Almeida et al., 2024).

Nutrition for pregnant sows must meet their own metabolic needs and support fetal growth. Reducing the protein level in the gestational diet may impair the reproductive performance of sows and the development of their fetuses, thereby affecting the growth and development of the offspring, because of the lack of limiting amino acids (Islas-Fabila et al., 2024). Lysine, methionine, tryptophan, and threonine are the limiting amino acids for swine under corn-soybean-based diets (Zhang et al., 2021). However, animals can synthesize nonessential amino acids (NEAA) through transamination. There is a report indicating that the nitrogen in the recommended diet for lactating sows based on NRC (2012) might be higher than the needs of sows (Spinler et al., 2024). Here, we proposed that appropriate reduction of dietary protein level in pregnant sows based on NRC (2012), in conjunction with the supplementation of limiting amino acids, may not impair the reproductive performance but reduce the nitrogen emissions.

## Materials and Methods

The animal study protocol was reviewed and approved by the Animal Care and Use Committee of Sichuan Agricultural University (20220132).

### Animals, feeding, and management

A total of 24 healthy Landrace × Yorkshire pregnant sows with similar body weight (BW) and backfat thickness (BF), in four to five parities, were randomly allocated to either the control group (CON, *n *= 12; BW 230.25 ± 3.73 kg, BF 15.49 ± 1.40 mm) or the LP group (LP, *n *= 12; BW 233.72 ± 5.57 kg, BF 16.13 ± 1.71 mm) on day 30 of gestation. After estrus synchronization, sows were artificially inseminated three times with semen from same Duroc boars. Gestating sows were fed with formulated corn-soybean based diets, with protein levels of 13.65% in the control group and 11.30% in the LP group from day 30 of gestation to the day before parturition. The other nutrients in the diet met the levels recommended for gestating sows by the National Research Council (NRC, 2012; Table 1). All sows were fed with the same lactation diet recommended by NRC (2012) during lactation (Table 2). Sows were housed in individual pens during gestation and lactation.

**Table 1.** Diet composition and nutrient levels during gestation of sows

Items	Control	Low-protein
**Ingredient, %**		
**Corn**	64.66	73.19
**Soybean meal**	14.00	7.00
**Wheat bran**	18.00	16.00
**L-Lysine HCl (98%)**	0.05	0.26
**D, L-Methionine (99%)**	0.02	0.05
**L-Tryptophan (98%)**	0.02	0.05
**L-Threonine (98.5%)**	0.05	0.15
**Limestone**	1.00	1.00
**Dicalcium phosphate**	1.30	1.40
**NaCl**	0.40	0.40
**Choline chloride (50%)**	0.15	0.15
**Vitamin and mineral premix^1^**	0.35	0.35
**Total**	100.00	100.00
**Nutrient levels^2^**		
**ME, Mcal/kg**	3.13 (3.14)	3.13 (3.16)
**CP, %**	14.00 (13.65)	11.00 (11.30)
**CF, %**	3.78	3.48
**Ca, %**	0.76	0.76
**AP, %**	0.41	0.40
**SID Lysine, %**	0.57	0.56
**SID Methionine, %**	0.24	0.24
**SID Threonine, %**	0.45	0.45
**SID Tryptophan, %**	0.13	0.13
**SID Arginine, %**	0.70	0.49
**SID Histidine, %**	0.37	0.30
**SID Valine, %**	0.54	0.43
**SID Phenylalanine, %**	0.51	0.40
**SID Leucine, %**	1.05	0.90
**SID Isoleucine, %**	0.42	0.31

Abbreviations: ME = metabolizable energy; CP = crude protein; CF = crude fiber; AP = available phosphorus; SID = standardized ileal digestible.

1 The premix provided each kilogram of diet: 120 mg Fe (FeSO_4_), 20 mg Cu (CuSO_4_), 120 mg Zn (ZnSO_4_), 30 mg Mn (MnSO_4_), 0.3 mg I (KI), 0.45 mg Se (NaSeO_3_), 12000 IU VA, 2400 IU VD_3_, 100 IU VE, 4.8 mg VK_3_, 2 mg VB_1_, 7.2 mg VB_2_, 3.6 mg VB_6_, 0.025 mg VB_12_, 40 mg niacin, 4 mg folic acid. The carrier of the premix is bran.

2 The Nutrient levels are calculated values. Measured values for metabolizable energy and crude protein level are shown in bracket.

**Table 2.** Diet composition and nutrient levels during lactation of sows

Ingredient	Content, %	Nutrient levels^2^	Content
**Corn**	63.38	ME, Mcal/kg	3.37
**Soybean meal**	22.20	CP, %	17.87
**Wheat bran**	6.00	CF, %	3.24
**Fish meal**	2.60	Ca, %	0.93
**Soybean oil**	2.00	AP, %	0.46
**L-Lysine HCl (98%)**	0.27	SID Lysine, %	1.04
**D, L- Methionine (99%)**	0.13	SID Methionine, %	0.41
**L-Tryptophan (98%)**	0.05	SID Threonine, %	0.56
**Limestone**	0.98	SID Tryptophan, %	0.20
**Dicalcium phosphate**	1.50	SID Tryptophan, %	0.71
**NaCl**	0.40	SID Valine, %	0.66
**Choline chloride (50%)**	0.15	SID Phenylalanine, %	1.33
**Vitamin and mineral premix^1^**	0.34	SID Leucine, %	0.59
**Total**	100.00	SID Arginine, %	1.00

1 The premix provides each kilogram of diet: 120 mg Fe (FeSO_4_), 20 mg Cu (CuSO_4_), 120 mg Zn (ZnSO_4_), 30 mg Mn (MnSO_4_), 0.3 mg I (KI), 0.45 mg Se (NaSeO_3_), 9600 IU VA, 1920 IU VD_3_, 80 IU VE, 3.8 mg VK_3_, 1.6 mg VB_1_, 5.8 mg VB_2_, 2.9 mg VB_6_, 0.02 mg VB_12_, 32 mg niacin, 3.2 mg folic acid. The carrier of the premix is bran.

2 The Nutrient levels are calculated values.

Sows were fed twice daily (08:30 and 14:30 h) with a total of 2.3 kg/day of either the control or LP diets from days 30 to 90 of gestation, and 2.72 kg/day from day 90 of gestation to farrowing. Sows were fed 1 kg/day of lactation diet on the first day of lactation, with the allowance increasing by 1 kg/day until day 6 of lactation, followed by ad libitum feeding from days 7 to 21 post-farrowing. Sows were individually housed in gestation crates and metabolic experiment was performed in metabolic cages (200 × 80 cm) during days 90 to 104 of gestation. On day 110 of gestation, sows were transferred to the farrowing house, where they were maintained under controlled environmental conditions with a temperature of 20–24°C and a humidity range of 50–65%. The number of piglets was adjusted to 12 ± 1 per litter (within the same treatment group) within 24 h postpartum. The sow milk was the sole food source for the nursing piglets during the lactation period. Weaning was conducted on day 21 of lactation.

### Sample collection and measurements

Feed samples during gestation were collected using the quartile method and stored at −20°C for subsequent analysis. The digestibility and metabolism experiment was performed from days 90 to 104 of gestation. Briefly, sows were transferred into metabolic cages on day 90 of gestation. After a 4-d adaptation, sows were fed with 0.5% iron oxide (Fe_2_O_3_) supplemented diets on day 5, and their respective diets for the other days. The feces were then collected from day 6 (when the reddish-brown color firstly appeared) to day 10 (when the reddish-brown color disappeared), and stored at −20°C for further analysis. At the same time, urine was collected with a urine basin from day 6 to 10 of the experiment. New urine basins with 60 mL of 10% sulfuric acid solution, which was used to fix nitrogen, were used to replace the old ones at 9:00 h every day. Of the daily urine, 50 mL was collected and stored at −20°C for subsequent analysis. In the morning of day 110 of gestation and day 21 of lactation, blood samples (10 mL) were obtained from the marginal ear vein under fasting condition. The blood samples were centrifuged at 3000×*g* for 15 min. The serum was collected and stored at −80°C for subsequent analysis.

At parturition, the total number of piglets born, the number of alive-born piglets, and the individual birth weight and body length were recorded immediately after delivery. Ten sows were randomly selected from each group immediately after parturition, and one male piglet with body weight closest to the litter average of each sow was selected for neonatal piglet sampling before nursing. The heart, liver, spleen, lung, kidney, pancreas, stomach, duodenum, jejunum, ileum, cecum (after removal of intestinal content), longissimus dorsi muscle, and placental samples were collected and weighed and were then immediately frozen in liquid nitrogen for subsequent analysis.

Samples of colostrum and mature milk were collected from the second and third mammary glands, with approximately 5 mL from each gland. Colostrum was collected within 3 h after farrowing. Mature milk was collected in the morning of days 14 and 21 of lactation before feeding. Prior to mature milk collection, sows were administered with oxytocin to facilitate milk let-down. The colostrum and mature milk were stored at −20°C for further analysis.

### Measurement of sow weight and backfat thickness

The body weight and backfat thickness of sows were recorded on days 30, 60, 90, and 110 of gestation, and days 1, 7, 14, and 21 of lactation, before feeding in the early morning. Backfat thickness was measured at the 10th rib on the left side 6.5 cm from the midline, with an ultrasound instrument (Renco Electronics, Manchester, MA).

### Tissue collection of weaned piglets

Ten sows were randomly selected from each group at weaning on day 21 of lactation, and one male piglet with body weight closest to the litter average of each sow was selected for weaned piglet sampling on the same day. After euthanasia, the heart, liver, spleen, lung, kidney, pancreas, stomach, duodenum, jejunum, ileum, and cecum (after removal of intestinal content) were collected and weighed. The lengths of the duodenum, jejunum, ileum, cecum, and longissimus dorsi muscle were measured. The cross-sectional area of the longissimus dorsi muscle was measured from the region between the 3rd and 4th ribs to the last rib. Organ indices were calculated for each organ.

### Determination of digestive metabolism

All the fecal samples collected from days 6 to 10 of digestibility and metabolism experiment of each sow were dried and mixed well. And all the urine samples collected from the digestibility and metabolism experiment of each sow were mixed well, followed by filtering with gauze.

The gross energy (GE) of the feed, feces, and urine samples was determined using a bomb calorimeter (model 6400; Parr Instrument Company, Moline, Illinois). The nitrogen contents in feed, feces and urine were measured using the Kjeldahl method (Morris et al., 2019). The following measurement formulas were employed:

Metabolizable energy (kcal) = ingested gross energy − fecal energy − urine energy.

Nitrogen deposition (g) = gross nitrogen intake − fecal nitrogen − urinary nitrogen.

Nitrogen deposition rate (%) = nitrogen deposition/gross nitrogen intake × 100%.

CP apparent digestibility (%) = (gross nitrogen intake − fecal nitrogen)/gross nitrogen intake × 100%.

Digestible energy (g) = gross energy − feces energy.

### Measurement of amino acids in serum and milk

Milk composition was analyzed using a Foss automatic milk analyzer (Fossomatic FC MilkoScan FT+; Foss Analytical, Suzhou, China), and the concentrations of free amino acids in the serum and milk were determined using an automatic amino acid analyzer (LA8080, Hitachi, Tokyo, Japan).

### RNA extraction and real-time PCR

The expression of genes associated with amino acid transporters was evaluated in placental samples. These genes included sodium-coupled neutral amino acid transporter 1 (SNAT1), sodium-dependent neutral amino acid transporter 2 (SNAT2), and sodium-coupled neutral amino acid transporter 4 (SNAT4). Glyceraldehyde-3-phosphate dehydrogenase (GAPDH) was selected as the endogenous reference gene. The primers for polymerase chain reaction (PCR) in this study are listed in Table 3.

**Table 3.** Primer sequences for real-time PCR

Gene	Primer sequence (5′–3′)	Accession number
***GAPDH***	F: ACACTGAGGACCAGGTTGTG	NM_001206359
	R: GACGAAGTGGTCGTTGAGGG	
***SNAT1***	F: TCAACGAGTCCTAACGCAACA	XM_003355629
	R: GGTAGCGCATACACGGTCTTT	
***SNAT2***	F: GGTGACTACTTGGTTCTGCTGG	NM_001317081
	R: AAGGAAAGGCCACTGGTGTATC	
***SNAT4***	F: GACTACACCCACCAGAAGCCT	XM_021092580
	R: CTTGTCATCACTGTGTGCTTCG	

### Statistical analysis

Data were analyzed using SAS 9.3 software (SAS Institute Inc., Cary, NC, USA). The normality and homogeneity of variances were verified using univariate procedures prior to the main analysis. *T*-test with Welch’s correction was used to compare the difference between two groups with normal distribution data, while non-Gaussian and heterogeneous data were analyzed using non-parametric Mann-Whitney test. Repeated measures ANOVA with post-hoc analysis was applied to analyze the repeatedly collected data, such as sow’s body weight, backfat thickness, milk composition, etc. Results are expressed as mean ± standard error (SE). Differences were considered significant at *P *< 0.05, and a trends was noted when 0.05 ≤ *P *< 0.10.

## Results

### Effects of low-protein diet during gestation on body weight and backfat thickness

The body weight of sows during gestation and lactation was unchanged by the LP diet, compared with the control diet (*P *> 0.05, Table 4). Besides, backfat thickness during gestation and lactation was similar between the two groups (*P *> 0.05). However, backfat thickness gain from day 30 to 60 of gestation was significantly lower in the LP group than that in the control group (*P *= 0.034, Table 5).

**Table 4.** Effect of gestational low-protein diet on body weight in sows

Body weight, kg	CON (*n *= 12)	LP (*n *= 12)	*P*-value		
			Diet	Time	Interaction
***Gestation***					
**Day 30**	230.25 ± 3.90^A^	237.24 ± 5.01^A^	0.046	<0.001	0.999
**Day 60**	239.53 ± 4.02^A^	247.78 ± 6.09^A^			
**Day 90**	249.18 ± 5.10^AB^	256.61 ± 5.32^AB^			
**Day 110**	267.23 ± 5.23^B^	274.30 ± 6.36^B^			
***Lactation***					
**Day 1**	239.48 ± 5.70	246.10 ± 6.13	0.282	0.306	0.968
**Day 7**	249.62 ± 5.23	255.12 ± 5.20			
**Day 14**	249.93 ± 4.89	251.66 ± 4.95			
**Day 21**	248.95 ± 5.85	251.86 ± 5.77			
***Weigh gain***					
**Days of G30–G60**	9.28 ± 2.13	10.53 ± 1.74	0.654		
**Days of G60–G90**	9.64 ± 1.99	8.83 ± 1.95	0.774		
**Days of G90–G110**	18.05 ± 1.02	17.69 ± 1.60	0.852		
**Days of G30–G110**	36.98 ± 3.11	37.06 ± 2.83	0.984		
**Days of G110–L1**	−27.74 ± 1.98	−28.20 ± 2.61	0.890		
**Days of L1–G7**	10.13 ± 3.46	9.02 ± 2.94	0.808		
**Days of L1–L14**	10.45 ± 4.03	5.56 ± 2.35	0.306		
**Days of L1–L21**	9.47 ± 4.18	5.76 ± 3.54	0.505		

Data are presented as mean ± SE.

A,B Different letters in same column of same period indicate significant difference. CON = control group; LP = low-protein group; G = gestation; L = Lactation.

**Table 5.** Effect of low-protein diet during gestation on backfat in sows

Backfat, mm	CON (*n *= 12)	LP (*n *= 12)	*P*-value		
			Diet	Time	Interaction
***Gestation***					
**Day 30**	15.49 ± 1.47	16.92 ± 1.73	0.879	0.997	0.936
**Day 60**	16.19 ± 1.58	16.28 ± 1.68			
**Day 90**	16.23 ± 1.76	15.83 ± 1.46			
**Day 110**	16.58 ± 1.79	16.17 ± 1.66			
***Lactation***					
**Day 1**	15.79 ± 1.71	15.33 ± 1.56	0.813	0.985	0.996
**Day 7**	15.63 ± 1.75	15.00 ± 1.58			
**Day 14**	15.29 ± 1.61	15.25 ± 1.57			
**Day 21**	14.92 ± 1.60	14.96 ± 1.67			
***Backfat gain***					
**Days G30–G60**	0.70 ± 0.36	−0.64 ± 0.47	0.034		
**Days G60–G90**	0.04 ± 0.48	−0.45 ± 0.54	0.508		
**Days G90–G110**	0.36 ± 0.27	0.34 ± 0.40	0.965		
**Days G30–G110**	1.09 ± 0.68	−0.75 ± 0.73	0.078		
**Days G110–L1**	−0.79 ± 0.35	−0.83 ± 0.29	0.927		
**Days L1–G7**	−0.17 ± 0.49	−0.33 ± 0.43	0.801		
**Days L1–L14**	−0.50 ± 0.57	0.08 ± 0.35	0.541		
**Days L1–L21**	−0.88 ± 0.55	−0.38 ± 0.63	0.556		

Data are presented as mean ± SE. CON, control group; LP, low-protein group; G, gestation; L, lactation.

### Effect of low-protein diet during gestation on sow reproductive performance

The farrowing performance of sows, including number, body weight, and body length of newborn piglets, were not affected by the LP diet compared with the control diet (*P *> 0.05, Table 6). Besides, the lactating performance of sows, including growth performance of piglets, feed intake of sows, composition of colostrum and milk, were similar between the LP and control groups (Tables 7–9).

**Table 6.** Farrowing performance of sows

Items	CON (*n *= 12)	LP (*n *= 12)	*P*-value
**Number of total born piglets, n**	17.17 ± 0.69	16.67 ± 0.96	0.676
**Number of alive born piglets, n**	14.83 ± 0.53	14.42 ± 0.73	0.541
**Number of stillbirths, n**	2.33 ± 0.58	2.25 ± 0.33	0.643
**Stillbirth rate, %**	12.87 ± 2.90	12.94 ± 1.68	0.985
**Litter weight at birth, kg**	20.30 ± 0.86	21.10 ± 1.28	0.608
**Alive litter weight at birth, kg**	17.94 ± 0.75	18.88 ± 0.98	0.457
**Average alive piglet weight at birth, kg**	1.26 ± 0.04	1.37 ± 0.06	0.177
**Average alive piglet length at birth, cm**	36.66 ± 0.34	37.18 ± 0.62	0.472

Data are presented as mean ± SE. CON, control group; LP, low-protein group.

**Table 7.** Growth performance of suckling piglets

Items	CON (*n *= 12)	LP (*n *= 12)	*P*-value		
			Diet	Time	Interaction
***Number of piglets***					
**Initial**	13.83 ± 0.44^A^	13.25 ± 0.46^A^	0.933	0.004	0.598
**Day 7**	12.78 ± 0.55^B^	12.33 ± 0.41^B^			
**Day 14**	11.78 ± 0.60^C^	12.25 ± 0.41^C^			
**Day 21**	11.56 ± 0.65^C^	12.00 ± 0.43^C^			
**Weaning survival rate, %**	83.87 ± 4.00	91.09 ± 2.88	0.157		
***Average body weight, kg***					
**Initial**	1.26 ± 0.04^A^	1.37 ± 0.06^A^	0.889	<0.001	0.940
**Day 7**	2.22 ± 0.09^B^	2.28 ± 0.10^B^			
**Day 14**	3.81 ± 0.20^C^	3.85 ± 0.18^C^			
**Day 21**	5.38 ± 0.35^D^	5.25 ± 0.33^D^			
**Days 1–21 weight gain**	4.13 ± 0.34	3.92 ± 0.33	0.576		
**Average body length at weaning^1^, cm**	55.34 ± 0.95	54.92 ± 1.18	0.784		
**Average carcass length at weaning^2^ ^,^ ^3^, cm**	36.13 ± 0.73	35.82 ± 0.83	0.784		

Data are presented as mean ± SE.

A,B Different letters in same column of same period indicate significant difference. CON = control group; LP = low-protein group.

1 Body length: the straight-line distance from the occipital crest to the coccygeal base.

2 Carcass length: the straight-line distance along the dorsal midline between the anterior edge of the first rib and the cranial border of the pubic symphysis.

3 *n *= 6 per group for the harvested piglets.

**Table 8.** Feed intake of sows during lactation

Duration	CON (*n *= 12)	LP (*n *= 12)	*P*-value		
			Diet	Time	Interaction
**Day 1–7, kg/d**	3.76 ± 0.11^A^	3.54 ± 0.18^A^	0.341	<0.001	0.710
**Day 8–14, kg/d**	6.83 ± 0.22^B^	6.36 ± 0.43^B^			
**Day 15–21, kg/d**	6.97 ± 0.27^B^	6.98 ± 0.39^B^			
**Day 1–21, kg/d**	5.90 ± 0.17	5.67 ± 0.31	0.516		

Data are presented as mean ± SE.

A,B Different letters in same column of same period indicate significant difference. CON = control group; LP = low-protein group.

**Table 9.** Components of colostrum and milk in sows

Items	CON (*n *= 12)	LP (*n *= 12)	*P*-value		
			Diet	Time	Interaction
***Fat content, %***					
**Day 0 of lactation (colostrum)**	5.26 ± 0.69^A^	5.87 ± 0.43^A^	0.538	<0.001	0.491
**Day 7 of lactation**	7.66 ± 0.50^B^	7.52 ± 0.38^B^			
**Day 14 of lactation**	7.80 ± 0.58^B^	7.14 ± 0.36^B^			
**Day 21 of lactation**	6.95 ± 0.32^B^	6.33 ± 0.31^B^			
***Crude protein content, %***					
**Day 0 of lactation (colostrum)**	17.77 ± 1.26^A^	17.80 ± 0.72^A^	0.896	<0.001	0.998
**Day 7 of lactation**	5.75 ± 0.15^B^	5.74 ± 0.08^B^			
**Day 14 of lactation**	5.49 ± 0.19^B^	5.37 ± 0.22^B^			
**Day 21 of lactation**	5.68 ± 0.20^B^	5.58 ± 0.27^B^			
***True protein content, %***					
**Day 0 of lactation (colostrum)**	16.58 ± 1.18^A^	16.68 ± 0.69^A^	0.939	<0.001	0.997
**Day 7 of lactation**	4.95 ± 0.14^B^	4.91 ± 0.08^B^			
**Day 14 of lactation**	4.66 ± 0.16^B^	4.56 ± 0.21^B^			
**Day 21 of lactation**	4.85 ± 0.19^B^	4.78 ± 0.25^B^			
***Lactose content, %***					
**Day 0 of lactation (colostrum)**	2.62 ± 0.13^A^	2.36 ± 0.14^A^	0.185	<0.001	0.294
**Day 7 of lactation**	5.54 ± 0.09^B^	5.72 ± 0.09^B^			
**Day 14 of lactation**	5.71 ± 0.09^B^	5.59 ± 0.15^B^			
**Day 21 of lactation**	5.63 ± 0.09^B^	5.27 ± 0.29^B^			
**Dry matter, %**					
**Day 0 of lactation (colostrum)**	29.15 ± 1.94^A^	29.23 ± 0.97^A^	0.473	<0.001	0.850
**Day 7 of lactation**	20.98 ± 0.60^B^	21.10 ± 0.48^B^			
**Day 14 of lactation**	20.99 ± 0.78^B^	20.11 ± 0.47^B^			
**Day 21 of lactation**	20.23 ± 0.52^B^	19.05 ± 0.51^B^			
***Urea nitrogen, mg/dL***					
**Day 0 of lactation (colostrum)**	79.55 ± 6.44^A^	76.78 ± 3.53^A^	0.228	0.001	0.917
**Day 7 of lactation**	63.22 ± 4.01^B^	62.57 ± 2.47^B^			
**Day 14 of lactation**	60.02 ± 2.40^B^	56.79 ± 2.38^B^			
**Day 21 of lactation**	63.95 ± 3.51^B^	58.19 ± 2.10^B^			

Data are presented as mean ± SE. CON = control group; LP = low-protein group.

^A,B^ Different letters in same column of same period indicate significant difference between time point.

### Effect of low-protein diet during gestation on organ indices in piglets

Maternal LP diet did not alter the organ indices in either neonatal or weaned piglets compared with the control diet (*P *> 0.05). Similarly, the relative length of the small intestine or the relative weight and length of the longissimus dorsi muscle were not altered by maternal LP diet, in either neonatal or weaned piglets (*P *> 0.05, Tables 10 and 11).

**Table 10.** Organ indices of neonatal piglets

Items	CON (*n *= 10)	LP (*n *= 10)	*P*-value
**Average piglet weight, g**	1269.90 ± 37.74	1320.00 ± 50.00	0.434
**Heart, ‰**	6.27 ± 0.19	6.43 ± 0.32	0.687
**Liver, ‰**	20.79 ± 1.14	20.82 ± 2.06	0.989
**Spleen, ‰**	0.81 ± 0.04	0.78 ± 0.04	0.586
**Lung, ‰**	12.97 ± 0.72	13.13 ± 0.53	0.860
**Kidney, ‰**	5.94 ± 0.38	6.43 ± 0.30	0.327
**Stomach, ‰**	4.63 ± 0.17	4.47 ± 0.18	0.544
**Pancreas, ‰**	0.87 ± 0.06	0.95 ± 0.04	0.277
**Duodenum, ‰**	0.65 ± 0.05	0.58 ± 0.03	0.159
**Jejunum, ‰**	22.63 ± 1.05	25.90 ± 1.78	0.131
**Ileum, ‰**	0.39 ± 0.03	0.40 ± 0.03	0.792
**Small intestine, ‰**	23.67 ± 1.09	26.87 ± 1.78	0.143
**Caecum, ‰**	0.53 ± 0.08	0.44 ± 0.05	0.312
**Carcass weight, ‰**	572.1 ± 8.13	564.43 ± 8.92	0.534
**Small intestine length, cm/kg BW**	239.46 ± 9.18	235.45 ± 13.44	0.808
**LD muscle length (Left), cm/kg BW**	11.28 ± 0.37	10.90 ± 0.46	0.530
**LD muscle index (Left), ‰**	8.97 ± 0.47	9.25 ± 0.50	0.691

Data are presented as mean ± SE. CON = control group; LP = low-protein group; BW = body weight; LD = longissimus dorsi.

**Table 11.** Organ indices of weaned piglets

Items	CON (*n *= 6)	LP (*n *= 6)	*P*-value
**Average piglet weight, g**	5392.50 ± 301.19	5460.78 ± 334.04	0.882
**Heart, ‰**	4.30 ± 0.22	4.52 ± 0.24	0.511
**Liver, ‰**	29.42 ± 1.71	26.29 ± 1.21	0.167
**Spleen, ‰**	1.74 ± 0.18	1.58 ± 0.11	0.493
**Lung, ‰**	10.87 ± 0.28	11.23 ± 0.24	0.349
**Kidney, ‰**	5.58 ± 0.45	5.71 ± 0.21	0.793
**Stomach, ‰**	4.71 ± 0.27	5.03 ± 0.26	0.411
**Pancreas, ‰**	1.24 ± 0.14	1.27 ± 0.08	0.892
**Duodenum, ‰**	0.77 ± 0.09	0.80 ± 0.13	0.820
**Jejunum, ‰**	36.13 ± 2.57	34.91 ± 2.59	0.745
**Ileum, ‰**	0.97 ± 0.09	1.07 ± 0.10	0.488
**Small intestine, ‰**	37.87 ± 2.68	36.78 ± 2.74	0.781
**Caecum, ‰**	1.36 ± 0.26	1.28 ± 0.120	0.781
**Carcass weight, ‰**	679.83 ± 14.54	665.37 ± 19.59	0.567
**Small intestine length, cm/kg BW**	119.88 ± 8.16	119.86 ± 6.89	0.999
**LD muscle length (Left), cm/kg BW**	4.18 ± 0.11	4.44 ± 0.23	0.344
**LD muscle index (Left), ‰**	14.67 ± 0.55	14.60 ± 1.06	0.954

Data are presented as mean ± SE. CON = control group; LP = low-protein group; BW = body weight; LD = longissimus dorsi.

### Effect of low-protein diet on nutrient digestion and metabolism in sows during late gestation

The LP diet significantly increased metabolizable energy intake, and reduced fecal energy and nitrogen levels compared with the control diet (*P *< 0.05). However, the nitrogen deposition rate of sows was unchanged by LP diet (*P *> 0.05, Table 12).

**Table 12.** Nutrient digestion and metabolism of sows during days 90 to 104 of gestation

Items	CON (*n *= 12)	LP (*n *= 10)^1^	*P*-value
**Gross energy intake, kcal/d**	10087.95 ± 18.83	10069.98 ± 31.00	0.619
**Fecal energy, kcal/d**	1370.74 ± 24.55	1199.25 ± 43.75	0.002
**Urine energy, kcal/d**	201.83 ± 20.17	172.68 ± 16.60	0.290
**Metabolizable energy intake, kcal/d**	8515.38 ± 33.36	8698.05 ± 49.29	0.005
**Digestible energy, g/d**	8717.21 ± 27.70	8870.73 ± 43.52	0.005
**Nitrogen intake, g/d**	59.19 ± 0.06	49.23 ± 0.01	0.000
**Fecal nitrogen, g/d**	7.37 ± 0.20	6.17 ± 0.30	0.002
**Urine nitrogen, g/d**	13.07 ± 1.29	10.07 ± 0.98	0.090
**CP apparent digestibility, %**	84.41 ± 0.24	86.38 ± 0.42	0.002
**Nitrogen deposition, g/d**	38.75 ± 1.30	32.99 ± 1.13	0.003
**Nitrogen deposition rate, %**	65.46 ± 2.16	67.01 ± 2.30	0.628

Data are presented as mean ± SE. CON = control group; LP = low-protein group.

1 Two sows in LP group were not performed with metabolic cage study due to lameness.

### Effect of low-protein diet during gestation on amino acid levels in serum and milk

In the pregnant sows, LP diet significantly decreased serum concentration of valine and serine (*P *< 0.05), and tended to decrease serum concentrations of arginine (*P *= 0.061), while tended to increase that of lysine. Serum concentrations of other amino acids were similar between the two groups (Table 13).

**Table 13.** Serum concentration of amino acids on day 110 of gestation in sows

Amino acid, nmol/mL	CON (*n *= 10)	LP (*n *= 10)	*P*-value
***Essential amino acids***			
**Arg**	77.28 ± 4.31	67.07 ± 2.73	0.061
**His**	48.37 ± 1.15	54.33 ± 3.50	0.122
**Ile**	49.98 ± 3.13	45.42 ± 3.17	0.319
**Leu**	76.82 ± 4.14	71.79 ± 4.35	0.427
**Lys**	50.19 ± 4.09	67.88 ± 9.26	0.098
**Met**	24.92 ± 0.83	24.78 ± 1.66	0.943
**Phe**	35.35 ± 1.35	35.65 ± 1.98	0.902
**Thr**	77.60 ± 3.86	91.02 ± 6.92	0.107
**Val**	92.62 ± 4.83	68.75 ± 3.96	0.001
**All EAAs**	533.13 ± 19.76	526.70 ± 27.94	0.853
***Nonessential amino acids***			
**Ala**	253.42 ± 12.50	268.13 ± 14.85	0.483
**Asp**	1.63 ± 0.40	1.74 ± 0.32	0.837
**Cys**	24.05 ± 1.33	22.91 ± 1.80	0.616
**Glu**	79.05 ± 5.80	89.07 ± 9.16	0.367
**Gly**	477.58 ± 16.11	451.01 ± 20.13	0.316
**Pro**	206.92 ± 9.48	219.77 ± 11.38	0.397
**Ser**	86.93 ± 3.18	76.98 ± 3.31	0.044
**Tyr**	38.85 ± 1.05	37.38 ± 2.70	0.619
**All NEAAs**	1168.43 ± 34.06	1166.98 ± 38.55	0.978

Data are presented as mean ± SE. CON = control group; LP = low-protein group.

In lactating sows, gestational LP diet decreased serum concentrations of arginine, lysine, methionine and glutamic acid compared to the control diet (*P *< 0.05), while tended to decrease serum concentrations of leucine (*P *= 0.095) and total EAAs (*P *= 0.053). Serum concentrations other amino acids remained unchanged by gestational LP diet (Table 14).

**Table 14.** Serum concentration of amino acids on day 21 of lactating in sows

Amino acid, nmol/mL	CON (*n *= 10)	LP (*n *= 10)	*P*-value
***Essential amino acids***			
**Arg**	81.47 ± 6.65	61.73 ± 3.71	0.004
**His**	46.65 ± 3.85	43.85 ± 3.16	0.581
**Ile**	56.65 ± 3.67	49.91 ± 5.11	0.298
**Leu**	103.07 ± 5.33	85.10 ± 8.71	0.095
**Lys**	92.72 ± 9.53	52.98 ± 8.29	0.006
**Met**	25.20 ± 1.91	18.72 ± 1.65	0.019
**Phe**	39.55 ± 2.50	37.1 ± 3.94	0.606
**Thr**	64.07 ± 6.77	50.01 ± 8.89	0.225
**Val**	122.12 ± 8.66	106.08 ± 11.08	0.269
**All EAAs**	631.49 ± 41.33	505.47 ± 44.46	0.053
***Nonessential amino acids***			
**Ala**	265.83 ± 17.24	261.18 ± 15.35	0.842
**Asp**	1.38 ± 0.14	1.23 ± 0.19	0.526
**Cys**	13.84 ± 0.87	14.70 ± 1.83	0.676
**Glu**	95.95 ± 5.48	115.07 ± 4.71	0.016
**Gly**	487.15 ± 26.30	506.63 ± 31.20	0.639
**Pro**	259.16 ± 18.11	229.94 ± 12.84	0.205
**Ser**	74.00 ± 5.30	69.38 ± 3.31	0.469
**Tyr**	39.80 ± 5.97	29.70 ± 3.86	0.173
**All NEAAs**	1237.12 ± 48.09	1227.81 ± 31.97	0.874

Data are presented as mean ± SE. CON = control group; LP = low-protein group.

LP diet during gestation decreased concentration of threonine in the milk compared to the control diet (*P *< 0.05), and tended to decrease that of isoleucine (*P *= 0.063), while those of other amino acids were similar between the two groups (Table 15).

**Table 15.** Amino acid contents in milk of sows on day 14 of lactation

Amino acid, nmol/mL	CON (*n *= 10)	LP (*n *= 10)	*P*-value
***Essential amino acids***			
**Arg**	66.87 ± 6.86	70.32 ± 8.74	0.760
**His**	11.42 ± 1.59	12.78 ± 1.37	0.524
**Ile**	5.26 ± 0.46	4.12 ± 0.34	0.063
**Leu**	22.79 ± 2.97	18.48 ± 1.94	0.240
**Lys**	40.52 ± 2.92	39.49 ± 5.15	0.863
**Met**	7.77 ± 0.83	7.09 ± 1.09	0.626
**Phe**	24.34 ± 3.22	22.52 ± 2.65	0.668
**Thr**	10.98 ± 2.15	5.26 ± 0.64	0.020
**Val**	26.46 ± 3.72	21.28 ± 4.08	0.360
**All EAAs**	218.90 ± 17.67	203.44 ± 20.35	0.573
***Nonessential amino acids***			
**Ala**	150.52 ± 20.04	149.26 ± 13.85	0.959
**Asp**	327.26 ± 21.88	291.36 ± 37.83	0.422
**Cys**	10.00 ± 1.03	8.08 ± 1.03	0.204
**Glu**	316.81 ± 23.04	289.28 ± 30.27	0.479
**Gly**	173.85 ± 20.10	176.24 ± 21.64	0.936
**Pro**	46.25 ± 4.78	35.88 ± 4.12	0.117
**Ser**	40.63 ± 3.26	39.43 ± 3.64	0.809
**Tyr**	26.54 ± 3.31	23.44 ± 3.09	0.503
**All NEAAs**	1091.86 ± 71.17	1012.97 ± 92.01	0.506

Data are presented as mean ± SE. CON = control group; LP = low-protein group.

Maternal LP diet decreased concentrations of leucine, methionine, and serine (*P *< 0.05), and tended to decrease concentrations of threonine (*P *= 0.050) and valine (*P *= 0.076) in the serum of neonatal piglets (Table 16). In the weaned piglets, serum concentration of threonine was decreased, while that of leucine was increased by maternal LP diet compared to control diet (*P *< 0.05, Table 17).

**Table 16.** Serum concentration of amino acids in neonatal piglets

Amino acid, nmol/mL	CON (*n *= 6)	LP (*n *= 6)	*P*-value
***Essential amino acids***			
**Arg**	56.25 ± 6.48	49.83 ± 10.03	0.602
**His**	44.46 ± 3.64	35.31 ± 4.33	0.137
**Ile**	53.31 ± 9.98	37.35 ± 6.96	0.219
**Leu**	83.58 ± 12.20	46.47 ± 7.08	0.025
**Lys**	131.67 ± 18.97	140.58 ± 19.72	0.752
**Met**	31.2 ± 3.49	19.71 ± 3.16	0.035
**Phe**	60.99 ± 9.67	48.72 ± 7.63	0.343
**Thr**	82.62 ± 8.87	59.40 ± 5.50	0.050
**Val**	168.33 ± 9.46	141.09 ± 9.98	0.076
**All EAAs**	712.59 ± 63.36	550.77 ± 31.73	0.050
***Nonessential amino acids***			
**Ala**	723.65 ± 30.36	604.73 ± 67.94	0.141
**Cys**	30.38 ± 4.28	23.45 ± 4.09	0.269
**Glu**	264.51 ± 31.41	229.59 ± 34.14	0.469
**Gly**	820.50 ± 66.57	725.05 ± 93.01	0.423
**Pro**	147.72 ± 6.46	133.71 ± 3.70	0.089
**Ser**	215.61 ± 6.42	154.65 ± 13.06	0.002
**Tyr**	72.48 ± 9.88	63.99 ± 10.43	0.568
**All NEAAs**	2225.01 ± 66.93	1828.98 ± 91.55	0.006

Data are presented as mean ± SE. CON = control group; LP = low-protein group.

**Table 17.** Serum concentration of amino acids in weaned piglets

Amino acid, nmol/mL	CON (*n *= 6)	LP (*n *= 6)	*P*-value
***Essential amino acids***			
**Arg**	100.03 ± 11.40	98.46 ± 6.95	0.909
**His**	86.31 ± 7.53	69.63 ± 5.46	0.103
**Ile**	48.96 ± 1.80	59.85 ± 6.41	0.133
**Leu**	82.38 ± 3.96	126.81 ± 5.82	0.000
**Lys**	180.06 ± 17.99	140.64 ± 21.13	0.186
**Met**	33.12 ± 6.25	35.13 ± 2.34	0.770
**Phe**	47.16 ± 6.63	47.78 ± 4.60	0.941
**Thr**	136.14 ± 8.10	65.78 ± 10.05	0.000
**Val**	136.08 ± 13.67	138.25 ± 13.44	0.912
**All EAAs**	850.24 ± 46.91	782.32 ± 52.74	0.359
***Nonessential amino acids***			
**Ala**	535.90 ± 62.09	489.63 ± 71.03	0.634
**Cys**	13.68 ± 0.67	15.15 ± 1.01	0.252
**Glu**	225.87 ± 34.23	155.05 ± 20.92	0.108
**Gly**	709.44 ± 83.02	716.22 ± 83.01	0.955
**Pro**	571.38 ± 25.58	432.85 ± 84.75	0.149
**Ser**	186.98 ± 24.13	160.98 ± 7.78	0.329
**Tyr**	64.71 ± 3.91	67.43 ± 8.86	0.785
**All NEAAs**	2307.95 ± 162.38	2037.30 ± 156.73	0.258

Data are presented as mean ± SE. CON = control group; LP = low-protein group.

### Low-protein diet during gestation did not alter the expression of amino acid transporter genes in the placenta of sows

**Figure 1.**
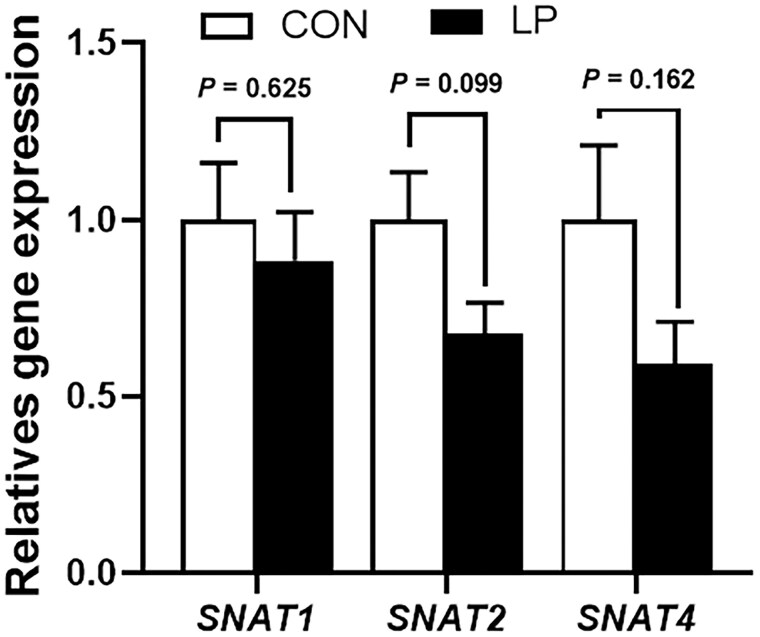
The expression of amino acid transporter genes in the placenta of sows. *N *= 6. CON, control group; LP, low-protein group.

The gene expression of amino acid transporter genes *SNAT1*, *SNAT2*, or *SNAT4* in the placenta of sows was not changed by the LP diet compared with the control diet (*P *> 0.05, Figure 1).

## Discussion

LP diets are adopted in the swine industry as a strategy for conserving protein feed. The current study showed that moderately reducing the dietary crude protein level (CP) during gestation (11.30% vs. 13.65%) reduced nitrogen excretion, without significantly impairing the reproductive performance in sows. These findings provide important evidence for the optimization of dietary protein levels and amino acid supplementation strategies for gestating sows.

Maternal dietary protein is used for fetal development and self-maintenance. The current study revealed that a LP diet (CP = 11.30% vs. 13.65%) during mid-to-late gestation did not alter the body weight and feed intake during gestation and lactation, but reduced backfat thickness gain from d 30 to 60 of gestation. Similarly, the study by Jang et al. (2014) revealed that sows with different dietary CP (11%, 13%, 15%, or 17%) and equivalent lysine level during gestation had similar body weight and backfat thickness changes during gestation and lactation. Eskildsen et al. (2020) also reported that LP diet during gestation (CP = 11.4% vs. 12.8%) did not alter the body weight or backfat thickness gain. However, another study revealed that a LP diet (CP = 10.53% vs. 12.82%) during gestation reduced body weight gain from d 90 to 110 of gestation without altering backfat thickness of sows (Yang et al., 2021). These studies indicate that an appropriate LP diet (CP no lower than 10.5%) supplemented with crystalline amino acids will not impair the growth performance of gestating sows.

Maternal dietary protein is important for fetal development and growth, as well as the reproductive performance (Xiao et al., 2025). The current study indicated that a LP diet (CP = 11.30% vs. 13.65%) fed to sows during mid-to-late gestation did not alter the number of alive born piglets or the body weight of alive piglets. Similarly, studies by Gajewczyk et al. (2010) (CP = 10.4%, 11.74%, or 13.01%), Fang et al. (2019) (CP = 10.5%, 12%, or 13.5%) and Kim et al. (2023) (CP = 11%, 12%, 13%, CP = 14%, 15% or 16%) revealed that sows fed with different dietary protein levels during gestation had comparable numbers of alive born piglets and piglet birth weight. Rachuonyo et al. (2002) also reported that a LP diet (CP = 12.6% vs. 14.7%) did not alter the number of alive born piglets and piglet birth weight. However, another study revealed that a LP diet (CP = 10.53% vs. 12.82%) during gestation increased numbers of total born piglets and stillborn piglets, without altering the number of alive born piglets and piglet birth weight (Yang et al., 2021). Of these studies, only Yang et al. (2021) used primiparous sows and found that a LP diet increased the number of stillborn piglets without altering the number of born alive piglets. These reports suggest that appropriate LP diets (CP no lower than 10.4%) supplemented with crystalline limiting amino acids may not impair the litter performance of multiparous sows. However, primiparous sows might be more sensitive to LP diets.

Gestation is a critical period for the formation and development of organs and muscles of fetus, particularly in the late stage of gestation (Feyera and Theil, 2017; Krogh et al., 2020). The present studies showed that reducing of dietary protein during mid-to-late gestation (CP = 11.30% vs. 13.65%) did not alter the birth weight, body length or organ indices, including weight of longissimus dorsi muscle, in newborn piglets. However, Wu et al. (1998) reported that dietary protein deficiency in sows could impair fetal growth and development. In the study by Kalbe et al. (2017), newborn piglets from sows fed with different protein levels during gestation (CP = 6.5% vs. 12.1%) had similar body weight. Another study revealed that a LP diet during mid-to-late gestation of sows (CP = 7.5% vs. 15%) did not change the birth weight of piglets (Jia et al., 2016). Fang et al. (2019) also found that a LP diet in gestating sows (CP = 10.5% vs. 13%) did not alter the birth weight or weaning weight of piglets. Furthermore, the study by Su et al. (2016) showed that low nutritional level during pregnancy impaired muscle growth and development in newborn piglets, but this could be recovered by supplementing of crystalline amino acids. Therefore, moderate LP diets supplemented with crystalline limiting amino acids during gestation (CP no lower than 10.5%) may not impair the development of fetus.

Adequate protein intake during pregnancy is crucial for the subsequent reproductive performance of sows; however excessive protein intake could increase nitrogen emission which may cause serious environmental pollution (Webb et al., 2014). The current study conducted a nitrogen metabolism experiment in sows during d 90 to 110 of gestation, and the results suggested that the LP diet during gestation (CP = 11.30% vs. 13.65%) significantly reduced fecal nitrogen excretion and nitrogen deposition in sows, while the nitrogen deposition rate was unchanged. Similarly, Galassi et al. (Galassi et al., 2010) reported that reduction of dietary CP by every 10 g/kg could decrease nitrogen excretion by approximately 8.0%. In another study, Yang et al. (2022) also observed that a LP diet (CP = 10.1% vs. 13.3%) in pregnant sows markedly decreased fecal nitrogen emissions without altering the nitrogen deposition rate. In the present study, the nitrogen deposition level decreased, but the nitrogen deposition rate did not increase, indicating that the ability of sows to deposit and utilize nitrogen was not affected. And, this was accompanied with the reduction of serum levels of arginine and valine in pregnant sows. The reduction of nitrogen deposition in the sows of LP group might because of the limitation of arginine and valine. The proportion of amino acids in the feed was reasonable to meet the physiological needs of sows, and the reduction of total nitrogen in the feed was the key factor in reducing nitrogen emissions (Kim et al., 2023). These studies indicate that moderate reduction of dietary protein levels with supplementation of crystalline limiting amino acids during pregnancy in sows could reduce nitrogen emissions.

Colostrum and milk are crucial for the growth and health of suckling piglets. The current study indicated that a LP diet (CP = 11.30% vs. 13.65%) in mid-to-late pregnant sows did not alter the levels of protein, fat and lactose in milk on d 7, 14, and 21 of lactation, accompanied by unaltered growth performance of suckling piglets. Similarly, previous studies showed that pregnant sows with different dietary protein levels had comparable milk concentrations of protein, fat and lactose, and growth performance of suckling piglets (Jang et al., 2014; Fang et al., 2019). These studies indicate that LP diets (CP no less than 10.5%) in pregnant sows may not impair their lactation performance and piglet growth performance.

Amino acids are extremely important for reproductive performance (Wu, 2009). The present study showed that the LP diet during gestation (CP = 11.30% vs. 13.65%) significantly reduced the serum valine level in sows on day 110 of gestation, consistent with our previous study, which reported significant lower serum valine concentration on days 30 and 90 of gestation in LP diet fed sows (CP = 10.1% vs. 13.3%) (Yang et al., 2022). Similarly, fattening pigs fed with LP diet (CP = 10% vs. 12% or 14%) had lower serum valine concentration (Duan et al., 2020). Valine is crucial for protein synthesis, blood glucose balance, immune function, and lactation ability (Zheng et al., 2016; Zhang et al., 2017; Wang et al., 2023). Actually, the standardized ileal digestible values of arginine (70%), histidine (81.1%), valine (79.63%), phenylalanine (78.43%), leucine (85.71%), and Ile (73.81%) were lower in the LP diet than those in the control diet. However, of the limiting amino acids, only valine was significant lower, and arginine trended to be lower in the serum of LP diet fed gestational sows than those in the NP group. What’s more, the serum levels of some limiting amino acids were even higher in the LP diet fed gestational sows than these in the control group, such as histidine, lysine and threonine, though no significant was reached. This might because lacking of serum valine and arginine in pregnant sows impaired the utilization of other amino acids in LP groups (Wang et al., 2023). And, this could be supported by the results that serum concentrations of almost all amino acids, including NEAAs, were lower in the neonatal piglets of LP group than those in the control group. Thus, valine and arginine should be supplemented in the low-protein diet of pregnant sows.

The current study showed that a maternal LP diet during pregnancy (CP = 11.30% vs. 13.65%) reduced serum concentrations of almost all amino acids in the newborn piglets, with the differences of concentrations of leucine, methionine, serine and total non-essential amino acids reached significance. However, the body weight and organ indices of newborn piglets were not altered by maternal LP diet. This might because the lack of valine and arginine in the serum of pregnant sows limited the transportation of amino acids through the placenta. However, this hypothesis should be confirmed in future study.

The current study also showed that a LP diet (CP = 11.30% vs. 13.65%) significantly decreased serum concentrations of arginine, methionine and lysine, and tended to reduce total essential amino acids in sows on d 21 of lactation, while their contents in the milk and serum of weaned piglets were comparable between the control and LP groups. Besides, the threonine contents in the milk and serum of LP group weaned piglets were lower than those of the control group, while serum concentration of threonine in LP group lactating sows was only 78% of that in control sows, although no significance reached. Milk secretion is a priority thing for lactating sows, and maternal protein is mobilized to synthesize milk protein (Zhang et al., 2018). The lower serum concentrations of arginine, methionine and lysine in LP group lactating sows might be attributed to reduced deposition of these amino acids during gestation, and some of them might be used to synthesis non-essential amino acids, though the standardized ileal digestible levels of lysine, methionine, threonine and tryptophan in feeds were similar between groups. Thus, the sows of LP group could not mobilize enough arginine, methionine, and lysine from body to synthesize milk protein compared with control group. This hypothesis will be confirmed in future study.

During lactation, with continued milk intake and internal nutrient deposition, serum amino acid levels gradually stabilize in the piglets of LP group, except for threonine. This might because the concentration of threonine in the milk of LP group was significantly lower than that of control group. Threonine is related to intestinal development and health (Mao et al., 2011). Thus, the decrease of threonine levels in milk and serum of weaned piglets may impair the post-weaning intestinal function and health. This hypothesis will be investigated in subsequent study.

A major limitation of the present study is that the sample size was small (12 sows per group, and serum samples for amino acids analysis were only 6 per group) This may reduce the statistical power and increase the risk of undetected biological differences. Our post-hoc analysis showed that the detection efficacy was moderate or low for some trends that did not reach statistical significance (such as the serum concentration of some essential amino acids). Despite of this limitation, the metabolism pattern of amino acids revealed in the present study consisted with the biological relevance.

In conclusion, moderate reduction of dietary protein level (11.30% vs. 13.65%) during mid-to-late gestation in sows could reduce their nitrogen excretion, while maintaining reproductive performance. In addition to lysine, methionine, threonine and tryptophan, arginine and valine might also be supplemented in LP diets to maintain the balance of serum amino acids in gestational sows. This study provides a basis for saving protein feed and reducing nitrogen excretion in pig industry.

## Supplementary Material

skaf412_Supplementary_Data

## Abbreviations:

AP: available phosphorus
BF: backfat thickness
BW: body weight
CF: Crudefiber
EAAs: essential amino acids
G: gestation
GAPDH: glyceraldehyde-3-phosphate dehydrogenase
L: Lactation
LD: longissimus dorsi
LP: low-protein group
ME: metabolizable energy
NEAAs: non-essential amino acids
PCR: polymerase chain reaction
SNAT1: sodium-coupled neutral amino acid transporter 1
SNAT2: sodium-coupled neutral amino acid transporter 2
SNAT4: sodium-coupled neutral amino acid transporters 4
SID: standardized ileal digestible
