# Supplementary material for: Effect of a low-protein diet during mid-to-late gestation on reproductive performance and serum amino acid profiles in sows
Source: J Anim Sci. 2025 Nov 29;103:skaf412. doi: 10.1093/jas/skaf412 (PMC12742152; doi:10.1093/jas/skaf412)
Supplement: skaf412_Supplementary_Data [file skaf412_supplementary_data.docx]

**Hightlights**

- A suitable low-protein diet in pregnant sows (CP=11.3% vs CP=13.65%) will not impair reproductive performance.
- Low-protein diet can reduce nitrogen emissions in pregnant sow.
- Except for Lys, Met and Trp, Arg and Val might also be required to be added in low-protein diet to keep the balance of serum amino acids in fetus.
- Thr is suggested to be added in lactating diet of sows which were fed with low-protein diet during gestation.
